# Therapeutic utility of immunosuppressive TREM2+ macrophages: an important step forward in potentiating the immune checkpoint inhibitors

**DOI:** 10.1038/s41392-020-00383-5

**Published:** 2020-11-10

**Authors:** Fulya Koksalar Alkan, Hasan Korkaya

**Affiliations:** 1 Research Institute for Health Sciences and Technologies, Istanbul Medipol University, Istanbul, Turkey; 2 Georgia Cancer Center, Department of Biochemistry and Molecular Biology, Medical College of Georgia, Augusta University, Augusta, GA 30912 USA

**Keywords:** Cancer microenvironment, Cancer therapy

**Fig. 1:**
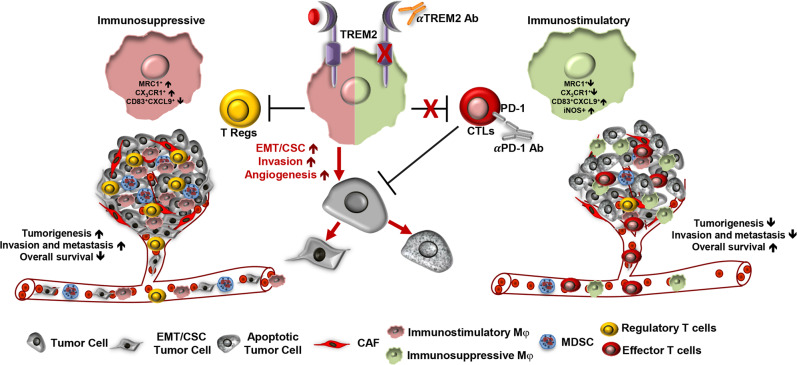
TREM2 modulated immunosuppressive tumor microenvironment is an attractive therapeutic target that also potentiates the efficacy of ICIs. Tumor-infiltrated TREM2+ macrophages, characterized by CX_3_CR1^hi^MRC1^low^CD83^low^CXCL9^low^ phenotype creates an TME which promotes aggressive properties on tumor cells while suppressing anti-tumor immune responses. Targeting the TREM2 by genetic ablation or anti-TREM2 antibody reverses the immunosuppressive TME and enhances the anti-tumor immune responses that eradicate tumors when combined with ICIs

In a recent article published in Cell, Molgora et al.^1^ reported that a subset of tumor-infiltrating macrophages with TREM2 expression creates an immunosuppressive microenvironment that promotes tumor growth while suppressing anti-tumor immune responses. Targeting these TREM2+ macrophages via genetic ablation of the gene or specific antibodies against the protein reduces tumor growth in animal models; however, it further attenuates tumor growth when combined with immune checkpoint inhibitors (ICI) by promoting the expression of immunostimulatory molecules (Fig. 1).

Immunosuppressive tumor microenvironment (TME) has long been implicated in therapeutic resistance; however, recent therapeutic advances using the ICI has magnified it’s importance.^2^ The clinical use of ICIs alone or in combination with other molecularly targeted therapeutics as first- or second-line therapy is currently leading the way in treatment of a plethora of malignancies. Although ICIs delivered promising durable responses in some patients with hard to treat malignancies, a majority of patients do not respond due to a wide range of resistance mechanisms. Furthermore, a fraction of ICI responders develops acquired resistance and progress to have a refractory disease. Tumor intrinsic mechanisms to ICIs such as the lack of checkpoint molecules, low levels of neoantigens, and inefficient antigen presentation due to downregulation or mutations of HLA genes have been well documented. However, accumulating evidence reveals a more complex mechanism of resistance to ICIs by immunosuppressive TME. In addition to their tumor-promoting activities by inducing neo-angiogenesis and tumor cell dissemination, accessory tumor-infiltrating myeloid cells such as macrophages, myeloid-derived suppressor cells (MDCS) and neutrophils have recently gained considerable attention due to their immunosuppressive capacity. Expectedly, immunosuppressive TME is associated with an advanced tumor stage and therapeutic resistance which determines the disease outcome.^3^ Therefore, it was proposed that targeting the immunosuppressive microenvironment might overcome resistance to the ICIs. Recent efforts in understanding of an iterative crosstalk between the myeloid cells, tumors, and cytotoxic lymphocytes have identified a number of therapeutic targets which are in pre-clinical or early phase clinical trials.

TREM2, a member of the transmembrane glycoprotein family, expressed on the surface of myeloid cells as well as on the microglia of the central nervous system and has been shown to play both immune and non-immune functions.^4^ While immune functions have been limited to restricting inflammation and phagocytosis of apoptotic debris, the functional importance of TREM2 in microglia of central nervous system (CNS) has been well characterized. Microglial TREM2 expression is shown to be elevated by several hundred-fold during a stroke and plays an important role in the protection of CNS from ischemic injury. Patients with dysfunctional TREM2 variants display progressive dementia and shorter life spans.

Existing literature provided a limited understanding of the significance of TREM2 in cancer that is primarily based on the correlation of TREM2 expression with clinical parameters such as the patient survival.^4^ Accumulating evidence suggested that elevated TREM2 expression correlated with tumor progression and poor patient survival in gastric cancer, glioma, and hepatocellular carcinoma. It is also important to note that some reports identified TREM2 as tumor suppressor based on its expression in malignant cells. Supporting the clinical significance of myeloid cells within the TME, we previously demonstrated that depletion of immunosuppressive MDSCs significantly reduces tumor growth and pulmonary metastasis in syngeneic mouse models.^5^ Furthermore, TREM2 regulates the expression of anti-inflammatory genes and antagonizes the pro-inflammatory responses which may be relevant in cancer development and progression. In line with these studies, TREM2-positive myeloid cells effectively inhibited T-cell proliferation.

Given that TREM2 is mainly expressed in myeloid cells which are the major components of the tumor microenvironment, Molgora et al.^1^ investigated the role of TREM2-positive macrophages in tumor development and progression. The team has first established the functional significance of TREM2 in cancer by using TREM2^−/−^ mice which significantly attenuated the growth of MCA-induced sarcoma, colorectal, and mammary tumors. Consistent with the notion that macrophages with high TREM2 expression predicted a poorer survival in patients with CRC and TNBCs. Investigators went on to demonstrate that a subset of TREM2 expressing myeloid cells played a crucial role in the formation of an immunosuppressive tumor microenvironment. The relative proportion of myeloid LY6C+MHCII^low/−^ subset was significantly reduced in tumors from TREM2^−/−^ mice in which the total CD11b^hi^ myeloid cell count has not changed compared to the wild type animals. Interestingly, tumors from TREM2^−/−^ mice displayed an increased tumor-infiltrating effector CD4^+^ and CD8^+^ T lymphocytes. The role of the latter was confirmed by the regrowth of the attenuated tumors upon specific depletion of CD8^+^ T cells. Thus, it was determined that reduced tumor growth in TREM2^−/−^ mice was dependent on generation of anti-tumorigenic TME and subsequent activation of cytotoxic T lymphocytes. A powerful single-cell RNAseq analyses of the immune infiltrates from tumors of WT and TREM2^−/−^ mice revealed eight different clusters of macrophages. Of these, CX_3_CR1-Macs and Cycling-Macs clusters were diminished, while Macs-1 subset, associated with IFNγ imprinting and immunostimulation, became the dominant cluster in TREM2^−/−^ mice. Furthermore, lymphoid landscape is also remodeled with enrichment of T cells and NK cells expressing activation markers IFNγ and PD-1 in TREM2^−/−^ mice bearing MCA tumors. These findings were also corroborated by using anti-TREM2 specific antibody with some nuanced differences. Anti-TREM2 treatment enriched iNOS+ macrophage cluster in MCA tumors in WT mice.

Collectively, their data suggested a potential utility of ICIs alone in TREM2^−/−^ mice or in combination with the anti-TREM2 antibody in WT animals bearing MCA tumors. Since the MCA tumors were immunogenic and responded to anti-PD-1 antibody, investigators used suboptimal dose of anti-PD-1 antibody and demonstrated that 100% of TREM2^−/−^ mice rejected MCA/1956 and MC38 tumors when treated with at early settings (3 days post-implantation) as well as the complete MCA tumor rejections in WT mice was confirmed when treated with the combination of anti-TREM2 and anti-PD-1 antibodies.

Although the clinical significance of immunosuppressive TME is firmly established, we still face significant challenges in developing adequate tools to effectively target these cells. Encouraging data presented in these studies provide a strong rationale in targeting these immunosuppressive cells in combination with standard of care therapeutics and ICIs.
